# Signaling pathways underlying nitrogen-dependent changes in root system architecture: from model to crop species

**DOI:** 10.1093/jxb/eraa033

**Published:** 2020-01-23

**Authors:** Zhongtao Jia, Nicolaus von Wirén

**Affiliations:** 1 Molecular Plant Nutrition, Leibniz Institute of Plant Genetics and Crop Plant Research (IPK), D-06466 Stadt Seeland, OT Gatersleben, Germany; 2 Nanjing Agricultural University, China

**Keywords:** Brassinosteroids, auxin, lateral root development, local signal, nitrate transporter, nitrogen signaling, nutrient efficiency, primary root development, root traits, systemic signal

## Abstract

Among all essential mineral elements, nitrogen (N) is required in the largest amounts and thus is often a limiting factor for plant growth. N is taken up by plant roots in the form of water-soluble nitrate, ammonium, and, depending on abundance, low-molecular weight organic N. In soils, the availability and composition of these N forms can vary over space and time, which exposes roots to various local N signals that regulate root system architecture in combination with systemic signals reflecting the N nutritional status of the shoot. Uncovering the molecular mechanisms underlying N-dependent signaling provides great potential to optimize root system architecture for the sake of higher N uptake efficiency in crop breeding. In this review, we summarize prominent signaling mechanisms and their underlying molecular players that derive from external N forms or the internal N nutritional status and modulate root development including root hair formation and gravitropism. We also compare the current state of knowledge of these pathways between Arabidopsis and graminaceous plant species.

## Teaser

Genetic factors determine how local and systemic nitrogen signals shape root system architecture in higher plants.

## Introduction

As a major constituent of biomolecules, nitrogen (N) has a strong impact on plant growth and development, in both natural and agricultural ecosystems. Plant roots preferentially take up the inorganic N forms NO_3_^–^ and NH_4_^+^, but also organic forms, including urea, amino acids, and peptides (Kojima *et al.*, 2007; Komarova *et al.*, 2008; Ganeteg *et al.*, 2017). The abundance of these N forms varies with soil type and climate. In well-aerated, neutral to high pH soils, nitrate is the most abundant N source, whereas ammonium can be a dominant N form in water-logged or low pH soils. In boreal and cold habitats, amino acids can account for a major fraction of the soluble N pool (Rothstein, 2009). The availability of different N forms in soils underlies considerable variation in space and time, as demonstrated by rapid turnover of amino acid-N and large concentration gradients of nitrate varying over one to several orders of magnitude (Lark *et al.*, 2004; Kielland *et al.*, 2007; Rothstein, 2009). Considering the sessile nature of plants, they must adapt continuously to spatiotemporal N fluctuations by sensing available N forms and modulating N transport or metabolism and, in the long run, their root system architecture to sustain plant growth.

Referring to root system architecture as the three-dimensional configuration of the root system, it is determined mainly by four parameters—growth, branching, surface area, and angle (Morris *et al.*, 2017). In dicotyledonous plants, such as *Arabidopsis thaliana*, the root system is composed of an embryonically formed primary root and individual post-embryonic lateral roots (Osmont *et al.*, 2007). In contrast, graminaceous species, such as maize or rice, form a fibrous root system composed of embryonic seminal roots, including eventually a primary root, as well as post-embryonic shoot-borne nodal or crown roots, which all undergo higher order lateral branching (Hochholdinger *et al.*, 2018). Despite these morphological differences, responses of many architectural traits of the two distinct root systems appear to be conserved at the genetic level (Rogers and Benfey, 2015). Such highly dynamic architectural responses of roots allow plants to optimize spatially defined soil exploration, improving plant performance under challenging N conditions. For instance, plant species forming more root biomass in deeper soil layers also deplete nitrate pools more efficiently (Heuermann *et al.*, 2019), and local proliferation of lateral roots into N-rich soil patches contributes significantly to plant N nutrition (Hodge *et al.*, 1999; Robinson *et al.*, 1999; Ma *et al.*, 2013). Under N-deficient conditions, plants develop a steeper root angle, which promotes deep rooting and facilitates N acquisition from subsoil layers, especially under soil conditions with high potential for nitrate leaching (Trachsel *et al.*, 2013; Saengwilai *et al.*, 2014). These observations imply that the simultaneous genetic improvement of root system architecture (RSA) and its plasticity to nutrient dynamics represents an effective strategy to improve N use efficiency. Here, we review the mechanisms by which external and internal N signals modulate root system architecture and, as far as possible, we compare these adaptive responses between the model species Arabidopsis and graminaceous crop species.

## Local N signals modulate lateral root elongation and branching

**Fig. 1.**
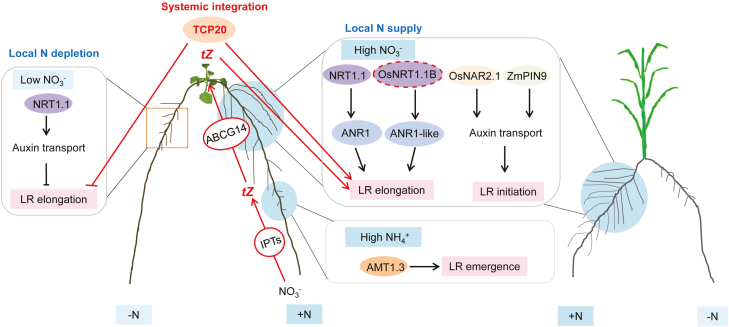
Local and systemic signaling involved in lateral root growth in response to local N supply in Arabidopsis and graminaceous species. In Arabidopsis, NRT1.1-dependent auxin removal from the lateral root (LR) primordium prevents LR elongation into N-depleted sites, while local high NO_3_^–^ promotes LR elongation involving the NRT1.1–ANR1 signaling pathway that is probably conserved in cereals. Local N signaling is integrated in the shoot via the transcription factor TCP20 and cytokinin (CK) signaling pathways. Local NH_4_^+^ stimulates LR emergence in an AMT1;3-dependent manner. OsNAR2.1 and ZmPIN9 modulate polar auxin transport that promotes LR initiation under local high NO_3_^–^ in rice and maize, respectively. The dashed outline indicates hypothetical functions or signaling steps that require further experimental validation*. tZ*, *trans*-zeatin; IPT, isopentenyl transferase.

Under natural conditions, soil is a spatially and temporally heterogeneous growth substrate for plant roots, in which a range of biotic and abiotic factors modulate the amount and distribution of different N forms. Precise proliferation of roots into N-rich soil zones is of crucial importance for plants to maximize soil exploitation for N. Plant strategies allowing an increase in foraging of localized N include the local stimulation of lateral root branching and elongation (Forde, 2014; Giehl *et al.*, 2014; Giehl and von Wirén, 2014; Liu and von Wirén, 2017; Fig. 1).

### Localized nitrate promotes lateral root elongation and density

When nitrate is locally supplied to an otherwise poorly N-supplemented root system, plants preferentially allocate root proliferation to NO_3_^–^-rich soil patches by stimulating lateral root elongation and/or branching; the extent of this response may vary across plant species (Drew *et al.*, 1975; Zhang and Forde; 1998; Remans *et al.*, 2006; Guan *et al.*, 2014; Yan *et al.*, 2014; Yu *et al.*, 2015*a*). There is plenty of experimental evidence that NO_3_^–^*per se* acts as a potent signaling molecule regulating lateral root proliferation in NO_3_^–^-rich patches (Zhang and Forde, 1998; Zhang *et al.*, 1999; Ruffel *et al.*, 2011). In Arabidopsis, detailed analyses in split-root growth systems revealed that adaptive responses of lateral roots to local NO_3_^–^ involves both local and systemic signals (Zhang and Forde, 1998; Zhang *et al.*, 1999; Remans *et al.*, 2006; Ruffel *et al.*, 2011; Guan *et al.*, 2014; Mounier *et al.*, 2014). Localized NO_3_^–^ modulates lateral root elongation by regulating meristematic activity in the lateral root tip via the nitrate transceptor NRT1.1/NPF6.3 (Zhang *et al.*, 1999; Remans *et al.*, 2006; Krouk *et al.*, 2010; Mounier *et al.*, 2014). In the absence of NO_3_^–^, NRT1.1/NPF6.3 acts as an NO_3_^–^-controlled auxin transporter that decreases auxin levels in the lateral root apex to suppress meristematic activity and lateral root elongation. This process acts upstream of the MADS-box transcription factor ANR1, which elicits a signaling cascade controlling lateral root elongation into zones of high NO_3_^–^ (Remans *et al.*, 2006; Bouguyon *et al.*, 2015, 2016). Information on local NO_3_^–^ availability is translocated to the shoot via long-distance mobile signals that include cytokinin (CK) and small peptides. In particular, *trans*-zeatin has been suggested to relay a long-distance systemic N demand signal that redirects lateral root growth from low-NO_3_^–^ to high-NO_3_^–^ zones (Ruffel *et al.*, 2011; Poitout *et al.*, 2018). In this case, local NO_3_^–^ provision stimulates isopentenyl transferase (IPT)-dependent *trans*-zeatin accumulation in the root and ABCG14-mediated root to shoot translocation (Ko *et al.*, 2014). The perception of *trans*-zeatin in the shoot then leads to transcriptional reprogramming and a shoot to root signal that induces nitrate transporters and root proliferation into the NO_3_^–^ patch. Remarkably, in the shoot, transcriptome genes involved in glutamate and glutamine biosynthesis were found to be significantly enriched, prompting a model in which shoot-derived amino acids may transmit CK-dependent N demand signals down to the roots (Kiba *et al.*, 2011; Poitout *et al.*, 2018). In a parallel signaling pathway, local N deprivation triggers synthesis of C-terminally encoded peptides (CEPs) in the root, which travel to the shoot via the xylem and are perceived by two leucine-rich repeat (LRR)-receptor kinases, CEP Receptor 1 and 2 (CEPR1/2; Tabata *et al.*, 2014). This triggers expression of two glutaredoxin-like small polypeptides, namely CEP Downstream1 and 2 (CEPD1/2), which are translocated to the roots and promote compensatory NO_3_^–^ uptake through up-regulation of the high-affinity NO_3_^–^ transporter gene *NRT2.1* in the NO_3_^–^-replete tissue (Ohkubo *et al.*, 2017). Although suggested, it remains elusive whether the CEP signaling pathway also regulates lateral root elongation in response to local NO_3_^–^. In addition, it has been shown that the transcription factor TCP20 functions as a systemic signaling regulator that directs lateral root foraging under heterogeneous NO_3_^–^ supply (Guan *et al.*, 2014). Thereby, TCP20 might act as an integrator of N and CK signaling, because this transcription factor can bind to promoters of type-A response regulators such as *ARR5/7* that are up-regulated by NO_3_^–^ in shoots (Ruffel *et al.*, 2011). Early genetic studies using abscisic acid (ABA) biosynthesis and signaling mutants suggested that ABA also participates in the lateral root elongation response to local NO_3_^–^ (Signora *et al.*, 2001). However, it still remains open how ABA regulates lateral root responses to localized nitrate. More recently, it has been shown that the mutant *abi2-2*, defective in the ABA co-receptor ABI2, phenocopies the attenuated lateral root response of the NRT1.1/NPF6.3 mutant allele *chl1-5* defective in nitrate perception. ABI2 modulates lateral root elongation possibly through interaction with CIPK23 and CBL1, because *abi2* mutant analysis and co-expression in oocytes showed that ABI2 largely decreased the phosphorylation state of CIPK23 and CBL1, which in turn stimulated NRT1.1/NPF6.3-dependent transport, and sensing and signaling of NO_3_^–^ (Leran et al., 2015). The CIPK/CBL proteins are major decoders of intracellular calcium (Ca^2+^) signatures (Dodd *et al.*, 2010). In this context, Riveras *et al.* (2015) proposed Ca^2+^ as a secondary messenger in the primary NO_3_^–^ response in Arabidopsis roots, raising the possibility that Ca^2+^ signaling exerts an early function in localized NO_3_^–^-induced lateral root elongation.

Despite the evolutionary distance between Arabidopsis and graminaceous plant species, lateral root growth in cereal crops is also strongly responsive to localized NO_3_^–^ (Wang *et al.*, 2002; Yu *et al.*, 2015*a*). In rice, local NO_3_^–^ supply promotes lateral root elongation and density in seminal roots, whereas in adult maize plants the extent of morphological plasticity of lateral roots depends on the root type and initiation time of shoot-borne roots (Wang *et al.*, 2002;  Yan *et al.*, 2014;  Yu *et al.*, 2015*a*). For instance, although local nitrate stimulates lateral root elongation primarily in embryonic primary and seminal roots, it can also increase the length and density of laterals on shoot-borne roots (Yu *et al.*, 2015*a*). In rice, evidence has been provided that ANR1-like MADS-box genes also regulate lateral root elongation in response to localized NO_3_^–^ (Yan *et al.*, 2014). The rice genome harbours five *ANR1*-like MADS-box transcription factor genes (*OsMADS23*, *25*, *27*, *57*, and *61*), four of which (*OsMADS23*, *25*, *27*, and *57*) are expressed in roots and are targets of the monocot-specific miRNA *miRNA444a* (Puig *et al.*, 2013; Yan *et al.*, 2014). Overexpressing *miR444a* in rice lowers expression of the target MADS-box genes and impairs their stimulatory effect on NO_3_^–^-dependent lateral root elongation (Yan *et al.*, 2014). This finding suggests that one or more of these *ANR1*-like genes play a similar role to that of Arabidopsis *ANR1* and that further *ANR1*-like genes may play evolutionarily conserved roles in NO_3_^–^-regulated lateral root elongation across dicots and monocots. Notably, here care should be taken as in rice *miRNA444a* has additional targets, whose activities have not yet been formally excluded as participating in lateral root adaptation to localized NO_3_^–^. More recently, the rice NO_3_^–^ transceptor NRT1;1B/OsNPF6.5, which is orthologous to AtNRT1;1/NFP6.3, has been discovered to control the natural genetic variation of N use efficiency between *indica* and *japonica* rice (Hu *et al.*, 2015). However, its role in NO_3_^–^-regulated lateral root growth and its potential link to *ANR1*-like genes are still unclear. In addition, another nitrate transporter in rice, OsNAR2.1, appears to modulate the responsiveness of lateral roots to localized nitrate (Huang *et al.*, 2015). In maize, increased growth rate and appearance of lateral roots under local NO_3_^–^ supply correlated positively with auxin levels in those root segments that were supplied with nitrate (Sattelmacher *et al.*, 1993). In accordance with distinct NO_3_^–^ responses of lateral roots in different root types, recent cell type-specific RNA sequencing approaches revealed root type-specific transcriptomes and a unique transcriptomic landscape in pericycle cells of brace roots (Yu *et al*., 2015*b*, 2016). Detailed analysis showed that the stimulatory effect of local NO_3_^–^ on lateral root initiation in shoot-borne roots depends on ZmPIN9-mediated auxin efflux and subsequent cell cycle activation facilitated by auxin/SCF^SKP2B^-mediated repression of Kip-related proteins (KRPs) (Yu *et al.*, 2015*b*).

### Local ammonium stimulates lateral root branching

NH_4_^+^ is a preferential N source for most crop plants (Gu *et al.*, 2013), and stimulation of lateral branching by local NH_4_^+^ has been observed for decades (Drew *et al.*, 1975; Hodge, 2004). Although enhanced lateral root proliferation under banded (i.e. localized) NH_4_^+^ supply is part of agricultural practice and contributes to enhanced fertilizer use efficiency (Ma *et al.*, 2013), it is not understood which mechanism underlies this adaptive response. A significant advance was made when Lima *et al.* (2010) discovered that local NH_4_^+^ and NO_3_^–^ act synergistically on lateral root proliferation and that in Arabidopsis NH_4_^+^-stimulated lateral root branching involves the NH_4_^+^ transporter AMT1;3. It was observed that NH_4_^+^-induced lateral root branching was significantly suppressed in the *amt1;3* mutant but almost absent in a quadruple NH_4_^+^ transporter mutant (*qko*; *amt1;1 amt1;2 amt1;3 amt2;1*). Interestingly, reconstituted expression of either *AMT1;3* or *AMT1;1* in *qko* showed that only AMT1;3 could restore lateral root proliferation, although both transporters share similar cell type-specific localization and NH_4_^+^ transport properties (Lima *et al.*, 2010). At that time, it remained open whether AMT1;3 acts as an NH_4_^+^ transceptor or whether the sensing event occurs downstream of AMT1;3, since AMT1;3 is consistently expressed in rhizodermal cells and rapidly feeds NH_4_^+^ into the symplast (Lima *et al.*, 2010; Duan *et al.*, 2018).

## Responses of root system architecture to homogeneous N signals

**Fig. 2.**
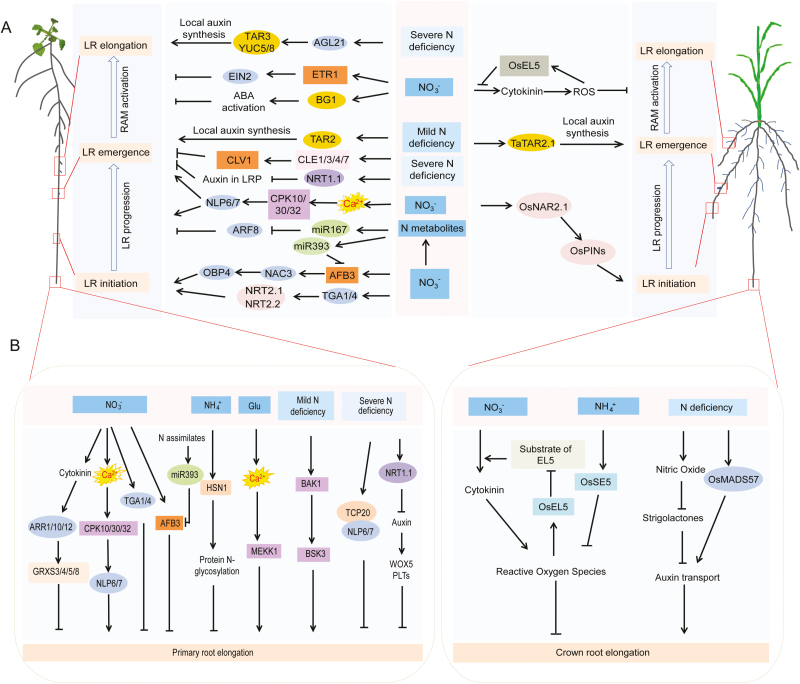
Signaling pathways shaping lateral root or primary root development in response to N deficiency or N supply in Arabidopsis and graminaceous species. (A) Signaling pathways involved in N-dependent lateral root (LR) formation in Arabidopsis and graminaceous species. Nitrate modulates LR growth in almost all developmental phases. Nitrate controls LR initiation through transcription factors TGA1/4 and the miR393/AFB3–NAC3–OBP4 signaling cascade. It also employs the miR167/ARF8 module and Ca^2+^–CPK10/30/32–NLP6/7 signaling to modulate LR progression/emergence. After the emergence, nitrate controls LR elongation through ETR1–EIN2-dependent ethylene and BG1-dependent ABA signalling pathways. In rice, OsNAR2.1 modulates polar auxin transport to control LR initiation, and OsEL5 interacts with NO_3_^–^-dependent cytokinin signaling to regulate LR elongation. Regarding N deficiency, severe N deficiency prevents LR emergence through the CLE/CLV1 peptide signaling module and NRT1.1-modulated auxin removal from LR primodia (LRP). It also positively regulates the MADS-box transcription factor AGL21 to regulate LR elongation, possibly through regulating local auxin biosynthesis. Mild N deficiency stimulates TAR2-dependent local auxin biosynthesis in the vasculature and pericycle to promote LR emergence, which is a rather conserved signaling cascade also discovered in wheat. (B) Signaling pathways shaping primary root development in Arabidopsis and graminaceous species under varying N availabilities. In addition to their roles in LR growth, transcription factors TGA1/4, miR393/AFB3, and Ca^2+^–CPK10/30/32–NLP6/7 signaling in Arabidopsis and OsEL5 in rice modulate primary root growth under nitrate supply. Nitrate also suppresses primary root elongation through glutaredoxins (GRXS3/4/5/8) acting downstream of cytokinin signaling. The inhibitory effect of ammonium on primary root elongation depends on HSN1-mediated protein *N*-glycosylation in Arabidopsis and production of ROS. In rice, OsSE5 can counteract the inhibition of ammonium by ROS detoxification. Glutamate (l-Glu) inhibits primary root elongation via the signaling kinase MEKK1. Severe N deficiency regulates root meristem size and distal stem cell differentiation, which involves TCP20–NLP6/7 and NRT1.1–auxin–WOX5/PLTs signaling pathways. In Arabidopsis, mild N deficiency enhances brassinosteroid signaling mediated by BAK1 and BSK3 to stimulate primary root elongation. In parallel, low N promotes crown root elongation in rice, which depends on polar auxin transport tuned by the intricate interaction of nitric oxide, strigolactones, and OsMADS57. ROS, reactive oxygen species; RAM, root apical meristem.

Root growth responses to homogenous NO_3_^–^ supply underlie dose-dependent regulation. Moderate NO_3_^–^ supply stimulates the growth of primary and lateral roots, whereas excess nitrate suppresses them (Zhang *et al.*, 1999; Vidal *et al.*, 2010; Liu *et al.*, 2017; Fig. 2). In addition to NO_3_^–^, evidence has been provided that NH_4_^+^ and the amino acid l-glutamate (l-Glu) inhibit root elongation (Walch-Liu *et al.*, 2006; Liu *et al.*, 2013; Fig. 2).

### The amount of homogeneously distributed nitrate modulates lateral root formation

Depending on the available concentration and growth context, NO_3_^–^ either stimulates or represses lateral root formation. These responses are orchestrated by spatial and temporal changes of gene expression in roots perceiving NO_3_^–^ signals (Gifford *et al.*, 2008; Walker *et al.*, 2017). Ca^2+^ signaling plays a central role in the stimulatory effect of NO_3_^–^ on lateral root formation, since intracellular NO_3_^–^ permeated by NRT1.1/NPF6.3 induces cytosolic Ca^2+^ waves that can be decoded by the subgroup III Ca^2+^-sensor protein kinases CPK10/30/32. These CPKs phosphorylate NIN-LIKE PROTEIN 6/7 (NLP6/7) to retain their nuclear localization, which promotes progression and emergence of lateral root primordia (Liu *et al.*, 2017). Excess NO_3_^–^ (≥10 mM) modulates phytohormone homeostasis and/or signaling to down-regulate most, if not all, developmental processes of lateral root formation (Zhang *et al.*, 2007; Krouk, 2016). In Arabidopsis, elevated NO_3_^–^ modulates lateral root formation via the auxin signaling modules *miR167*/*ARF8* and *miR393*/*AFB3* (Gifford *et al.*, 2008; Vidal *et al.*, 2010). Cell type-specific transcriptome profiling revealed that downstream of NO_3_^–^, the N metabolites glutamine/glutamate repress the expression of *miR167* in pericycle cells, allowing accumulation of *ARF8* transcripts in the pericycle to control the key developmental checkpoint between lateral root initiation and emergence (Gifford *et al.*, 2008). Unlike ARF8 that is not regulated by NO_3_^–^, NO_3_^–^*per se* strongly induces expression of the auxin receptor gene *AFB3*, whose transcript levels are feedback repressed by downstream N assimilates via *miR393* that targets the *AFB3* transcript for degradation (Vidal *et al.*, 2010). Mutation of *AFB3* or overexpression of *miR393* markedly abrogates the stimulatory effect of NO_3_^–^ on lateral root initiation (Vidal *et al.*, 2010). This pathway has been further extended by the finding that the transcription factor gene *NAC4* and its target *OBP4* act downstream of *AFB3* to specifically promote lateral root initiation and emergence (Vidal *et al.*, 2013). Using a systems biology approach, the two bZIP transcription factor genes *TGA1* and *TGA4* have been identified. TGA1/4 act downstream of the nitrate transceptor NRT1.1/NFP6.3 and bind to promoters of the two high-affinity NO_3_^–^ transporter genes *NRT2.1/NRT2.2* to regulate lateral root initiation (Alvarez *et al.*, 2014).

After emergence from the parental root, the meristem of lateral roots is activated to commence elongation. Excess supply of NO_3_^–^ (≥10 mM) interferes with this developmental process by inducing a systemic signal to repress the elongation of lateral roots (Zhang *et al.*, 1999). Analysis of ABA biosynthesis and signaling mutants suggested the involvement of ABI4- and ABI5-dependent ABA signaling as well as of an ABA-independent pathway mediating this systemic repression (Signora *et al.*, 2001). Recently, NO_3_^–^ provision (30 mM) has been shown to stimulate the release of ABA from a conjugated form (ABA-glucose) by the induction of a β-glucosidase (BG1), thereby eliciting downstream an ABA response that represses lateral root growth (Ondzighi-Assoume *et al.*, 2016). Exposure to high NO_3_^–^ (10 mM) rapidly induces ethylene production and represses lateral root growth. Lateral root growth in *etr1-3* and *ein2-1* mutants, both impaired in ethylene signaling, is insensitive to high NO_3_^–^, suggesting a regulatory role for ethylene in the systemic repression of lateral root growth by high NO_3_^–^ (Tian *et al.*, 2009). Compared with these extensive studies in Arabidopsis, it is poorly understood how excess NO_3_^–^ regulates lateral root branching in crop plants, except for the characterization of OsEL5, a ubiquitin ligase that interacts with NO_3_^–^-dependent cytokinin signaling to repress lateral root formation (Mochizuki *et al.*, 2014).

### Effects of homogeneously supplied nitrate on primary root elongation

Apart from lateral roots, NO_3_^–^ also regulates primary root elongation. The auxin signaling module *miRNA393*/*AFB3* and the transcription factors TGA1/4 have been shown to control the inhibitory effect of NO_3_^–^ on primary root elongation, while the signaling cascade NO_3_^–^–Ca^2+^–CPKs–NLP6/7 promotes primary root growth (Vidal *et al.*, 2010; Alvarez *et al.*, 2014; Liu *et al.*, 2017). Thereby, a link has been established between glutaredoxins and NO_3_^–^. NO_3_^–^ up-regulates expression of a group of glutaredoxin genes (*AtGRXS1/3*/*4*/*5/6*/*8*/*11*) in shoots, while RNA silencing of *AtGRXS3/4/5/8* reverses the inhibitory effect of elevated NO_3_^–^ on primary root growth (Patterson *et al.*, 2016). NO_3_^–^-dependent up-regulation of *AtGRXS3/4/5/8* appears to be mediated by CKs, because this response becomes almost completely lost in an *arr1,10,12* mutant that is defective in CK signaling (Patterson *et al.*, 2016). These results collectively suggest that NO_3_^–^ provision stimulates biosynthesis of CKs in roots and their translocation to the shoot, where CKs activate *ARR1/10/12*-mediated expression of *GRX* genes, which may be transported rootwards to repress primary root elongation. Likewise, in primary, seminal, and crown roots of maize, high NO_3_^–^ (≥10 mM) inhibits cell elongation, which coincides with decreased auxin levels in apical root segments (Tian *et al.*, 2008). Physiological approaches further suggest auxin, CKs, and nitric oxide (NO) to trigger NO_3_^–^-inhibited root elongation (Tian *et al*., 2005, 2008; Zhao *et al.*, 2007); however, the underlying molecular mechanisms remain to be discovered.

### Elevated ammonium inhibits root elongation

Whenever NH_4_^+^ is supplied to plants as the sole N source, it inhibits the elongation of primary and lateral roots. Evidence from supplying NH_4_^+^ to different root zones suggested that NH_4_^+^ is sensed in the root apex (Li *et al.*, 2010). At the cellular level, NH_4_^+^ considerably represses cell proliferation and expansion (Liu *et al.*, 2013). In an earlier study, auxin has been suggested to be involved in NH_4_^+^-mediated inhibition of root elongation, because mutants impaired either in auxin transport (*aux1*) or in signaling (*axr1* and *axr2*) are more resistant to NH_4_^+^ (Cao *et al.*, 1993). However, a more recent study showed that in the presence of NH_4_^+^, primary root elongation of *aux1* is still as sensitive as in the wild type (Liu *et al.*, 2013), leaving it open whether auxin participates in NH_4_^+^-dependent inhibition of root elongation. One component in the molecular mechanism underlying NH_4_^+^-inhibited primary root elongation has been unmasked by the isolation of the *hsn1-1* mutant that is hypersensitive to NH_4_^+^ (Qin *et al.*, 2008). *HSN1*, allelic to *VTC1*, encodes a GDP-mannose pyrophosphorylase (GMPase). The lack of HSN1 and subsequent *N*-glycosylation of proteins has been associated with root hypersensitivity to NH_4_^+^ (Qin *et al.*, 2008; Barth *et al.*, 2010). NH_4_^+^ triggers the production of reactive oxygen species (ROS) that act as signaling molecules in primary root elongation (Patterson *et al.*, 2010; Tsukagoshi *et al.*, 2010; Bloch *et al.*, 2011). In rice, the heme–heme oxygenase OsSE5, a novel antioxidant regulatory enzyme, is strongly induced by elevated NH_4_^+^, while inhibition of root elongation by NH_4_^+^ is markedly mitigated by up-regulation of OsSE5 activity using a chemical heme oxygenase inducer or by overexpression of *OsSE5* in Arabidopsis or rice (Xie *et al.*, 2015). It has been reported that ROS levels do not differ between wild-type and *vtc1* mutant plants, suggesting that GDP-mannose-dependent protein *N*-glycosylation and ROS signaling act independently in the regulation of primary root elongation in the presence of NH_4_^+^. In addition, a mutant defective in *Indeterminate Domain 10* (*IDD10*) was also shown to be hypersensitive to NH_4_^+^, while the detailed mechanism is unknown (Xuan *et al.*, 2013).

### **l**-**Glutamate as a signal suppressor of primary root growth**

Although NO_3_^–^ and NH_4_^+^ are the predominant N forms in most soils, organic N forms can also account for a major fraction of the available N pool, for instance in bog soils, and interfere with root growth processes. Previous work has shown that even very low concentrations (≤0.05 mM) of external l-Glu can remarkably inhibit primary root elongation and stimulate root branching, consequently resulting in a shorter but more branched root system (Walch-Liu *et al.*, 2006). The sensing of l-Glu appears to take place in the root tip, because the inhibitory effect of l-Glu supplied locally to primary root tips was as strong as that supplied to the whole root system. In this case, NRT1;1/NPF6.3-dependent NO_3_^–^ signaling could antagonistically suppress l-Glu-mediated inhibition of primary root growth (Walch-Liu and Forde, 2008). With respect to auxin, l-Glu has been shown to reduce expression of the auxin-responsive reporter DR5–GFP (green fluorescent protein) in the primary root apex, while in auxin mutants (*aux1-*7, *axr1-3*, and *axr1-12*), the sensitivity of primary root growth to l-Glu is altered (Walch-Liu *et al.*, 2006). However, the exact role of auxin in l-Glu-mediated root architectural changes remains to be demonstrated. Regarding the mode of action, l-Glu-elicited signal transduction involves the mitogen-activated protein (MAP) signaling kinase MEKK1 (Forde *et al.*, 2013). Very recently, quantitative trait locus (QTL) analysis using recombinant inbred lines derived from reciprocal crosses between the Arabidopsis accessions C24 and Col-0 mapped a major locus, named *GluS1*, controlling root sensitivity to l-Glu (Walch-Liu *et al.*, 2017). Future studies isolating the causal gene are required to extend our understanding on the mechanistic action by which l-Glu inhibits primary root growth.

## Dual effects of N deficiency on root system architecture

A systematic comparison of root architectural changes under diverse nutrient deficiencies revealed a dose dependency of root architectural traits on external N supply and on the plant N nutritional status (Gruber *et al.*, 2013; Fig. 2). Thereby, mild and severe N deficiency can be distinguished by shoot fresh weight and shoot N concentration responses to external N, which both decreased between 550 µM and 275 µM N supply, indicative of mild N deficiency, while at ≤100 µM external N (i.e. severe N deficiency), shoot N concentrations dropped below the critical deficiency level (Gruber *et al.*, 2013). Accordingly, root growth was stimulated in a concentration range of 200–550 µM N, whereas at <100 μM external N, plants adopt a ‘survival strategy’ by inhibiting the elongation of both primary and lateral roots as well as the emergence of new lateral roots (Gruber *et al.*, 2013; Giehl and von Wirén, 2014). This morphological response restricts root growth to nutritionally unfavorable environments to economize on the cost for root development in favor of plant survival. In contrast, at external N levels of 200–550 µM that induce mild deficiency, plants expand their root system by increasing the emergence of lateral roots (Ma *et al.*, 2014; Shao *et al.*, 2017) and in particular the length of primary and lateral roots (Chun *et al.*, 2005; Gruber *et al.*, 2013; Sun *et al.*, 2014). This foraging response enhances the capacity of the root system to explore deeper soil horizons where N may be more abundant.

### Severe N deficiency restricts root branching and elongation

Under persistent N deficiency, early lateral root development is modulated by a regulatory pathway that involves CLAVATA3/ESR-related (CLE) signaling peptides and their receptor protein, the LRR receptor-like kinase CLAVATA1 (CLV1) (Araya *et al*., 2014*a*, *b*, 2016). While expression of several *CLE* homologs, including *CLE3*, is up-regulated by low N (≤0.1 mM) in pericycle cells, the receptor protein CLV1 localizes to phloem companion cells (Araya *et al.*, 2014*a*). Whether this indicates CLE peptide cycling via the shoot or radial short-distance transport within the root remains open. Focusing on lateral root primordia, N deprivation favors the accumulation of NRT1.1/NFP6.3, which facilitates the shootward movement of auxin, thereby lowering auxin accumulation in lateral root primordia to inhibit their emergence (Krouk *et al.*, 2010; Bouguyon *et al*., 2015, 2016). Low nitrate further modulates stem cell dynamics in the root meristem with profound impacts on root system architecture. TCP20, a systemic master regulator in nitrate signaling, physically interacts with NLP6/7 to regulate the expression of the cell cycle gene *CYCB1;1* and cell division in the root apical meristem (Guan *et al.*, 2017). In addition to cell division, low nitrate (0.05 mM) is also found to promote differentiation of distal stem cells (Wang *et al.*, 2019). At low nitrate, when auxin accumulation in the root apex is significantly suppressed, expression levels of the stem cell identity genes *WOX5* and *PLT1/2* become down-regulated. This response involves NRT1.1/NFP6.3, because roots of *chl1-12* plants, defective in NRT1.1/NFP6.3 function, are less sensitive to low N in terms of distal stem cell differentiation and show higher expression levels of *WOX5* and *PLT* genes. These observations led to a working model, in which low N restricts auxin accumulation in the root tips via NRT1.1/NFP6.3-dependent signaling and subsequently represses expression of *WOX5* and *PLT* genes to promote distal stem cell differentiation. Considering that in the context of low nitrate, NRT1.1/NFP6.3 prevents auxin accumulation in the lateral root primordium via its auxin transport activity (Krouk *et al.*, 2010), distal stem cell differentiation is more likely to be due to the activity of NRT1.1/NFP6.3 in auxin transport rather than in nitrate sensing. Further analyses on distal stem cell differentiation in *chl1-9* or T101A/D substitution lines that can decouple nitrate sensing from auxin transport will refine the mechanism by which NRT1.1/NFP6.3 modulates stem cell differentiation (Bouguyon *et al.*, 2015). N deprivation also induces expression of the MADS-box transcription factor gene *AGL21* (Yu *et al.*, 2014). Mutation in *AGL21* leads to impaired lateral elongation especially under severe N limitation, probably through modulated local auxin biosynthesis.

### Mild N deficiency stimulates root elongation and branching

The stimulation of root growth by mild N deficiency is of particular interest as it increases the soil volume that can be explored for nutrient acquisition. The positive effect of mild N deficiency on lateral root formation requires the auxin biosynthesis gene *TAR2*. The corresponding protein catalyzes the first step in the main auxin biosynthesis route by converting tryptophan to indole-3-pyruvic acid (Zhao, 2012; Ma *et al.*, 2014). Mild N deficiency induces expression of *TAR2* in the pericycle and vasculature of mature root zones, whilst *tar2* mutants show reduced lateral root numbers especially under mild N deficiency (Ma *et al.*, 2014). So far, it is less likely that TAR2-mediated auxin biosynthesis is also necessary for lateral root elongation, because TAR2-dependent auxin production cannot explain the phenotype of lateral root elongation. Recently, Brumos *et al.* (2018) showed that precise spatial expression of auxin biosynthesis genes is critical for root development and root responses to ethylene. It is very likely that the inability of TAR2-dependent auxin biosynthesis to modulate lateral root elongation is due to its spatially restricted expression in the root maturation zone (Ma *et al.*, 2014; Ursache *et al.*, 2014). More recently, a role for brassinosteroid (BR) signaling has been discovered to regulate root foraging under mild N deficiency. By employing genome-wide association mapping, Jia *et al.* (2019*a*) identified *Brassinosteroid Signaling Kinase 3* (*BSK3*) as being associated with a QTL of primary root length and promoting cell elongation in response to mild N deficiency. Interestingly, allelic variation caused by a single amino acid substitution from proline to leucine enhances plant BR sensitivity and signaling, which increases the root foraging response. Whereas N deficiency has no impact on the transcriptional regulation of *BSK3*, transcript levels of the BR co-receptor gene *BAK1* increase under N deficiency, suggesting systemic N deficiency signals entering BR signaling in roots via *BAK1* (Jia *et al.*, 2019*a*). Like Arabidopsis, crop plants also develop longer roots under N deficiency, albeit with considerable genotypic variation (Chun *et al.*, 2005; Sun *et al.*, 2014; Melino *et al.*, 2015; Shao *et al.*, 2017). Although BR signaling components have been well characterized in cereals, for example in rice, maize, and barley, their roles in modulating root foraging under N deficiency await discovery (Dockter *et al.*, 2014; Kir *et al.*, 2015; Tong and Chu, 2018). In rice, a combined role for strigolactones and auxin has been implicated in low N-induced seminal and adventitious root elongation (Sun *et al.*, 2014). It has been reported that low N enhances strigolactone biosynthesis and signaling, involving the genes *D10*, *D27*, and *D3*, which in turn reduces OsPIN1b-mediated rootward polar auxin transport and attenuates seminal root growth (Sun *et al*., 2014, 2018). Upstream of strigolactone signaling, low nitrate stimulates NO production. NO targets the strigolactone signaling repressor D53 for degradation, thereby allowing elongation of seminal and adventitious roots (Sun *et al.*, 2016). Most recently, OsMADS57 has been shown to modulate seminal and adventitious root elongation under low nitrate by modulating PIN-directed auxin accumulation in the root tips (Huang *et al.*, 2019). So far, it has been shown that OsMADS57 modulates tillering in rice through D14-dependent strigolactone signaling (Guo *et al.*, 2013). The role of OsMADS57 in strigolactone-modulated polar auxin transport and in root elongation, however, awaits further investigation.

An inconsistency across plant species has been observed regarding the regulation of lateral root density by low N. On the one hand, it has been reported that low N increases lateral root emergence in Arabidopsis and wheat (Ma *et al.*, 2013; Shao *et al.*, 2017). On the other hand, low N decreased lateral root density in rice and maize (Sun *et al.*, 2014; Gao *et al.*, 2015). As mentioned above, low N stimulates vasculature-expressed *TAR2* to promote lateral root emergence in Arabidopsis (Ma *et al.*, 2014). A conserved function of its orthologous gene *TaTAR2.1* has been reported in wheat, in which transgenic lines with reduced or enhanced expression grow fewer or more lateral roots, respectively, under low N (Shao *et al.*, 2017). In rice, low nitrate elevates strigolactone levels and decreases lateral root density via D3, an F-box protein mediating strigolactone perception (Sun *et al.*, 2014). Furthermore, knockdown of *OsNAR2.1*, encoding a partner protein of the high-affinity nitrate transporters OsNRT2.1/2.2/2.3a, inhibits lateral root formation in response to nitrate, probably through decreased PIN-mediated rootward transport of auxin (Huang *et al.*, 2015).

## **Signaling mechanisms underlying N**-**dependent root hair formation and root gravitropic responses**

Root hairs greatly expand the absorptive surface area of roots and express a number of nutrient transporters, which facilitates the uptake of water and minerals. The formation and elongation of root hairs are highly responsive to environmental N signals, indicating that root hair growth can be seen as a read-out for N-sensing events. Mechanisms shaping N-dependent root hair plasticity are just beginning to emerge. In a recent study, high NO_3_^–^ availability has been shown to increase root hair density mainly by suppressing the longitudinal elongation of trichoblasts (Canales *et al.*, 2017). The mechanism of action involves the NO_3_^–^ transceptor NRT1;1/NPF6.3 and the signal transducers TGA1/4, which directly regulate expression of the root hair-specifying gene *CPC* to increase root hair density. In the case of NH_4_^+^, the tonoplast-localized Ca^2+^-associated protein kinase CAP1 has been proven essential for NH_4_^+^-regulated root hair growth (Bai *et al.*, 2014). In this case, CAP1 acts as modulator of cytoplasmic NH_4_^+^ homeostasis that establishes tip-focused cytoplasmic Ca^2+^ and pH gradients as prerequisite for root hair growth. Regardless of N forms, root hair length is continuously increasing with decreasing N levels in the growth substrate (Vatter *et al.*, 2015). Auxin and ethylene have been shown to play key roles in root hair formation and to be responsible for root developmental changes elicited by nutrient signals (Tian *et al.*, 2009; Bhosale *et al.*, 2018). To what extent these phytohormones also modulate root hair elongation under N-deficient conditions remains to be discovered. Although a number of regulators of root hair development have been isolated in crops, including rice and maize (Giri *et al.*, 2018; Hochholdinger *et al.*, 2018), it remains completely unknown which mechanisms mediate root hair responses to varying N availabilities in cereal crops.

Root growth angle is critical for the spatial distribution of the root system in soils and thus for nutrient and water uptake. For instance, it has been shown that the steeper root growth angle conferred by *DRO1* in rice plants increases tolerance to drought (Uga *et al.*, 2013). Regarding responses of the root growth angle to N availability, it has been observed that moderate levels of NH_4_^+^ increase gravitropism while excess NH_4_^+^ causes agravitropism (Zou *et al.*, 2012). Notably, this phenomenon is independent of the inhibitory effect of NH_4_^+^ on root elongation (Liu *et al.*, 2013). NH_4_^+^-induced agravitropism is associated with repression of the auxin efflux carrier PIN2 and subsequent failure of asymmetric auxin distribution in the root elongation zone (Zou *et al.*, 2012; Liu *et al.*, 2013). In addition, *Altered Response to Gravity 1* (ARG1) has been shown to sustain PIN3-mediated lateral auxin gradients across the lateral root cap and AUX1-facilitated basipetal auxin movement to antagonize the role of PIN2, thereby protecting root gravitropism under excess NH_4_^+^ (Zou *et al.*, 2013). In response to low N, the root growth angle becomes steeper, probably for improved N foraging in deeper soil layers (Trachsel *et al.*, 2013). However, the underlying mechanisms are still unknown.

## **N**-**dependent root architectural changes dependent on other nutrient signals**

**Fig. 3.**
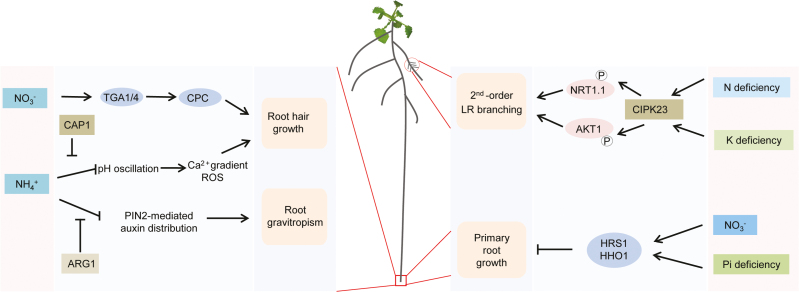
Nitrogen-coordinated root hair growth, gravitropism, and root architectural changes by interaction with other nutrient signals in Arabidopsis. Nitrate activates a signaling cascade involving TGA1/4 and CPC to increase root hair density. Ammonium induces intracellular pH imbalance, which disturbs cytosolic Ca^2+^ gradients and activates reactive oxygen species (ROS), leading to swelling of root hairs. This process can be alleviated by a tonoplast-localized receptor kinase CAP1, which maintains intracellular ammonium homeostasis by vacuolar compartmentation. In addition, ammonium inhibits PIN2-mediated asymmetric auxin distribution and prevents root gravitropism, which can be counteracted by ARG1. CIPK23 is an integrator of low N–K signals into the regulation of second-order lateral root (LR) branching. This process requires the nitrate transceptor NRT1.1 and potassium channel AKT1; both are phosphorylation targets of CIPK23. Nitrate also coordinates the primary root response to phosphate deficiency by regulating the activity of HRS1/HHO1.

Although extensive studies have been carried out to elucidate how roots respond to N signals, phenotypic changes in root traits depend not only on the supplied N but also on the level of supply of other nutrients (Fig. 3). Indeed, this is an expected scenario for plant roots growing in natural heterogeneous soils, where roots are exposed to multiple facets of nutrient gradients at the same time. Recent exciting studies reveal intricate interactions between NO_3_^–^ and phosphate (Pi) signaling in shaping plant physiological and developmental processes (Medici *et al*., 2015, 2019; Hu *et al.*, 2019). The reduction in primary root elongation under Pi deficiency depends on NO_3_^–^ signalling. In this case, the GRAS family transcription factor gene *HRS1* together with its paralog *HHO1* have been reported to be transcriptionally induced by NO_3_^–^ but post-translationally regulated by Pi starvation before coordinating an NO_3_^–^-dependent primary root response to Pi deficiency (Medici *et al.*, 2015). Evidence also points to crosstalk between N and potassium (K) signaling. For example, primary root elongation is strongly repressed under low K, which in turn depends on NH_4_^+^ (Xu *et al.*, 2006). Another angle of N–K interaction appears with regard to the modulation of lateral root branching. It shows that low NO_3_^–^ can largely suppress second-order lateral root formation induced by K starvation (Kellermeier *et al.*, 2014). Analysis of root systems in mutants defective in genes with known roles in K and NO_3_^–^ transport and/or signaling suggests crucial roles for the NO_3_^–^ transceptor NTR1.1/NPF6.3 and the K channel AKT1 in this root response (Kellermeier *et al.*, 2014). In this case, CIPK23 appears to be a connective node integrating NO_3_^–^ and K signaling through phosphorylation of NTR1.1 and AKT1 to regulate lateral root formation (Tsay *et al.*, 2011; Kellermeier *et al.*, 2014).

## Conclusion and perspectives

Over the past couple of years, several root responses to N have been investigated across plant species and found to be highly conserved. Significant progress has been made in elucidating the molecular and cellular basis of how roots sense environmental N signals, in particular by the discovery of phytohormones and peptides conveying local and systemic N signals in the model plant Arabidopsis (Tabata *et al.*, 2014; Ohkubo *et al.*, 2017; Poitout *et al.*, 2018; Jia *et al.*, 2019*a*). In crops, however, much work remains to be done as compared with the knowledge gained in Arabidopsis, as most studies in graminaceous species do not go far beyond the stage of physiological description. Particularly successful examples, such as modulating the expression of Arabidopsis-orthologous *ANR1*-like genes in rice or *TAR2* in wheat (Yan *et al.*, 2014; Shao *et al.*, 2017), indicate conserved roles for these genes in N-driven root architectural changes, underscoring the possibility to translate findings from Arabidopsis to crop species. Thus, ongoing efforts in characterizing orthologous genes in cereals will further advance our understanding of N sensing and downstream root responses in crops. With regard to recent studies showing that root responses to N are integrated with responses to other environmental stimuli (Kellermeier *et al.*, 2014; Medici *et al*., 2015, 2019), future challenges will lie in the identification of regulators intersecting these responses. In particular, genomic studies in combination with systems biology approaches have great potential to uncover the most relevant players in the corresponding regulatory networks. There is a large intraspecific variation in root system architectural traits in Arabidopsis as well as in crop species (Rosas *et al.*, 2013; Maccaferri *et al.*, 2016; Jia *et al.*, 2019*b*). Obviously, with the development of high-throughput, non-destructive root phenotyping platforms and advanced imaging tools (Kenobi *et al.*, 2017), genome-wide association studies will become an even more powerful tool for decoding the genetic complexity of RSA responses to N signals and the identification of useful natural alleles. Carried further, identified favorable alleles can be used for root tissue- or cell type-specific expression and allele-specific modification in crops, taking advantage of CRISPR/Cas9 technology for the benefit of improved N use efficiency.
